# Between a hygiene rock and a hygienic hard place: Avoiding SARS-CoV-2 while needing environmental exposures for immunity

**DOI:** 10.1093/emph/eoab006

**Published:** 2021-02-12

**Authors:** William Parker, Joshua T Sarafian, Sherryl A Broverman, Jon D Laman

**Affiliations:** 1 Department of Surgery, Duke University Medical Center, Durham, NC, USA; 2 Department of Biology and the Duke Global Health Institute, Duke University, Durham, NC, USA; 3 Department of Biomedical Sciences of Cells and Systems, University Medical Center Groningen, Groningen, The Netherlands

**Keywords:** COVID-19, SARS-CoV-2, hygiene, evolutionary mismatch

## Abstract

Suboptimal understanding of concepts related to hygiene by the general public, clinicians and researchers is a persistent problem in health and medicine. Although hygiene is necessary to slow or prevent deadly pandemics of infectious disease such as coronavirus disease 2019 (COVID-19), hygiene can have unwanted effects. In particular, some aspects of hygiene cause a loss of biodiversity from the human body, characterized by the almost complete removal of intestinal worms (helminths) and protists. Research spanning more than half a century documents that this loss of biodiversity results in an increased propensity for autoimmune disease, allergic disorders, probably neuropsychiatric problems and adverse reactions to infectious agents. The differences in immune function between communities with and communities without helminths have become so pronounced that the reduced lethality of severe acute respiratory syndrome coronavirus 2 in low-income countries compared to high-income countries was predicted early in the COVID-19 pandemic. This prediction, based on the maladaptive immune responses observed in many cases of COVID-19 in high-income countries, is now supported by emerging data from low-income countries. Herein, hygiene is subdivided into components involving personal choice versus components instituted by community wide systems such as sewage treatment facilities and water treatment plants. The different effects of personal hygiene and systems hygiene are described, and appropriate measures to alleviate the adverse effects of hygiene without losing the benefits of hygiene are discussed. Finally, text boxes are provided to function as stand-alone, public-domain handouts with the goal of informing the public about hygiene and suggesting solutions for biomedical researchers and policy makers.

Lay Summary: Hygiene related to sewer systems and other technology can have adverse effects on immune function, and is distinct from personal hygiene practices such as hand washing and social distancing. Dealing with the drawbacks of hygiene must be undertaken without compromising the protection from infectious disease imposed by hygiene.

## INTRODUCTION

Coronavirus disease 2019 (COVID-19) is an infectious disease caused by severe acute respiratory syndrome coronavirus 2 (SARS-CoV-2), a single stranded RNA virus in the Coronaviridae family. In the first year following the World Health Organization’s declaration of the disease a Public Health Emergency of International Concern on 30 January 2020, the disease claimed >2 million lives worldwide. Much of the reported mortality was centered in particular high-income countries. For example, based on data collected by the Johns Hopkins Coronavirus Resource Center as of January 2021, the USA and the UK together accounted for more than one quarter of all fatalities during the first year, despite accounting for only about 5% of the world’s total population.

During the first months of the COVID-19 pandemic, data began to emerge connecting adverse and often deadly reactions to the SARS-CoV-2 virus with autoimmune-like reactions (reviewed by Halpert and Shoenfeld [1]). Based on that information, and based on the known impact of the loss of symbiotic intestinal worms (helminths) associated with hygiene, one of us made a prediction in May of 2020, posted on social media, that infection with the SARS-CoV-2 virus may not be as deadly in parts of the world without widespread use of toilets and water treatment facilities (www.facebook.com/WilliamParkerLab, last accessed December 17, 2020). This prediction is now supported by several comparative studies examining the impact of COVID-19 in different parts of the world [2–6].

In this review, we discuss hygiene and its multiple impacts on human health. Particular regard is given to the impact of hygiene on the COVID-19 pandemic. On the one hand, hygiene helps alleviate very high burdens of infectious disease that result from increased population densities that, in turn, occurred as a result of the development of agriculture and urban centers. For example, fortunately, hygiene measures help reduce the morbidity and mortality resulting from the COVID-19 pandemic. On the other hand, hygiene causes loss of some species from the ecosystem of the human body, which in turn leads to susceptibility to a range of chronic inflammatory disease. Unfortunately, this susceptibility to chronic inflammatory disease is apparently associated with adverse reactions to a large number of viral pathogens, probably including SARS-CoV-2.

In the first section of this review, a brief history of our understanding of the impact of hygiene on human health is provided. Hygiene will be divided into two categories based on how hygiene is implemented in society and based on the impact of that hygiene. Next, the impact of the two types of hygiene on the COVID-19 pandemic will be discussed. Finally, we will discuss potential solutions to the problem as well as hurdles that currently impede implementation of those solutions. Text boxes summarizing this information are provided to function as stand-alone, public-domain handouts, both for informing the general public about the importance of hygiene and to provide useful information for biomedical research scientists and policy makers involved with our response to the COVID-19 pandemic.

## THE DARK SIDE OF HYGIENE

Although it is widely known that hygiene must be employed to slow or in some cases prevent pandemics of infectious disease, not all effects of hygiene are positive. The term hygiene hypothesis was first coined by David Barker in 1988, who observed that improved sanitation conditions are associated with appendicitis [7]. A year later, David Strachan used the term again, suggesting that increased exposures to a wide range of pathogens might decrease the likelihood of allergic disease [8]. The hygiene hypothesis was derided at the time because no plausible mechanism that might explain the hypothesis had been discovered [9]. However, both Barker and Strachan’s work proved to be seminal and is now understood in terms of improved immune regulation as a result of exposure to particular types of organisms such as intestinal worms, called helminths [10–12].

Unknown to Barker and Strachan when they published their studies, Brian Greenwood, more than a decade earlier, had hypothesized that infections with helminths and protists could explain the lack of autoimmune disease he observed in Ibadan, Nigeria [13, 14]. Greenwood quickly went on to demonstrate that a mild infection with protists, which produced no apparent long-term adverse effects, completely rescued laboratory mice that would otherwise die from a lupus-like autoimmune condition [15]. At the same time, again via exposure to protists, Greenwood was able to block autoimmune disease in a laboratory rat model of rheumatoid arthritis [16]. Soon afterward, Peter John Preston observed that exposure to helminths effectively alleviates symptoms of seasonal allergies [17]. Preston’s observation was confirmed by a self-infection experiment conducted by Jon Turton, described in 1976 [18]. More recently, studies in animal models by Maizels *et al.* [19, 20] as well as prospective clinical studies by Correale *et al.* [21–23] have extended the apparently beneficial role of exposure to helminths to include prevention of autoimmune conditions [24, 25]. Further, emerging evidence indicates that exposure to helminths also helps prevent or alleviate a range of inflammation-associated neuropsychiatric disorders, including chronic fatigue syndrome, anxiety disorders and major depressive disorders [26–29].

At the present time, the state of our understanding can be described as a Biota Alteration Theory, whereby a hygiene-induced lack of biodiversity in post-industrial society, including the essentially complete loss of complex eukaryotic symbionts such as helminths and protists, poses a major evolutionary mismatch [30, 31]. This evolutionary mismatch, or point of incompatibility between our biology and modern society, creates generalized immune dysfunction and predisposes humans to a wide range of inflammation-associated maladies [30–33]. This adverse result of the loss of complex eukaryotic symbionts can be readily understood in light of the hundreds of millions of years of vertebrate evolution in the presence of helminths [34]. In short, the vertebrate immune system has evolved in the presence of helminths and protists, and as a result is now dependent on exposure to these organisms for effective development and function [35].

Although hygiene causes one evolutionary mismatch by eliminating symbiotic organisms that are necessary for efficient immune function, it is widely appreciated that hygiene alleviates the consequences of another evolutionary mismatch. In particular, hygiene, in combination with vaccine programs, mitigates the high burdens of infectious disease that result from crowded, urban environments. These crowded environments and their associated burden of infection constitute an evolutionary mismatch that is very distinct from but yet tied closely to the evolutionary mismatch of biota alteration.

## PERSONAL HYGIENE VERSUS SYSTEMS HYGIENE

Importantly, the seminal work by Greenwood, Preston, Turton, Barker, Strachan, Maizels, Correale and others that led to our current understanding was associated with a type of hygiene that is no longer thought of as hygiene, per se, in most parts of post-industrial, high-income countries. Hygiene today in high-income countries is not defined by whether we use a toilet, whether we have refrigeration and plastic storage containers, whether our food is harvested and prepared using modern technology, or whether we have water from municipal sources. These factors create a type of hygiene, which we will label systems hygiene. This type of hygiene is indeed necessary to prevent the spread of infectious disease and also causes biota alteration and the ensuing propensity for chronic, non-infectious and inflammation-associated diseases. But this is not that type of hygiene that those of us in industrial societies tend to think about when the term hygiene is mentioned.

**Figure 1.**
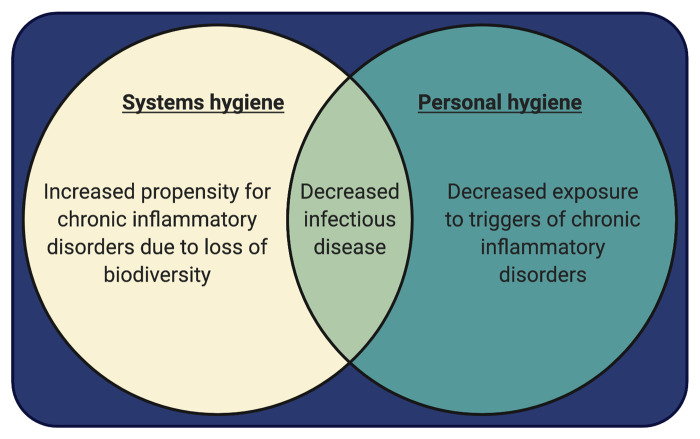
Two types of hygiene with sometimes overlapping and sometimes opposing effects. Triggers of chronic inflammatory disorders that are reduced by personal hygiene include viral infections, dust mite derived allergens and mold

When people think of hygiene in high-income countries, they think of personal hygiene, the type of hygiene that is *not* at the root of biota alteration. Personal hygiene is well recognized by washing hands, brushing teeth, taking showers and appropriate social distancing when needed. This type of hygiene, as with systems hygiene, is necessary for the prevention and control of pandemics of infectious disease. However, unlike systems hygiene that leads to biota depletion and subsequent chronic inflammatory disease, personal hygiene actually decreases acute inflammatory stressors such as mold, insect-derived and mite-derived allergens and acute viral infections, all of which can trigger chronic inflammatory disease in an immune system destabilized by biota alteration. Thus, personal hygiene, unlike systems hygiene, can reduce the burden of chronic, non-infectious, inflammation-associated disease. A summary of the two types of hygiene and their corresponding effects are shown in Fig. 1 and in Box 1.

As shown in Fig. 1 and summarized in Box 1, it is not tenable to compensate for biota alteration by eliminating either systems hygiene or personal hygiene. Elimination of either type of hygiene would result in catastrophic morbidity and mortality from infectious disease. Further, relaxing personal hygiene would likely only increase exposure to triggers of chronic inflammatory disease without alleviating the biota alteration induced by systems hygiene.

## THE IMPACT OF BIOTA ALTERATION ON DISEASE AND THE COVID-19 PANDEMIC

The effects of biota alteration began to emerge in the late 19^th^ century and continue to expand to this day. Some of the first effects were documented toward the latter part of the 19^th^ century and include seasonal allergies [36] and appendicitis [37]. Although the pathological effects of biota alteration can be diverse, they share several factors in common. The diseases tend to be chronic, difficult to cure using pharmaceutical approaches and are always associated with inflammation. Further, although the evolutionary mismatch of biota alteration predisposes individuals to chronic inflammatory disease, the induction of disease usually depends on a wide range of other factors, including genetics and exposure to triggers that can induce disease. For example, while biota alteration is at the root of seasonal allergies [17], such allergies are associated with genetic predisposition as well as exposure to allergens such as ragweed pollen and dust mite-derived proteins.

**Figure 2.**
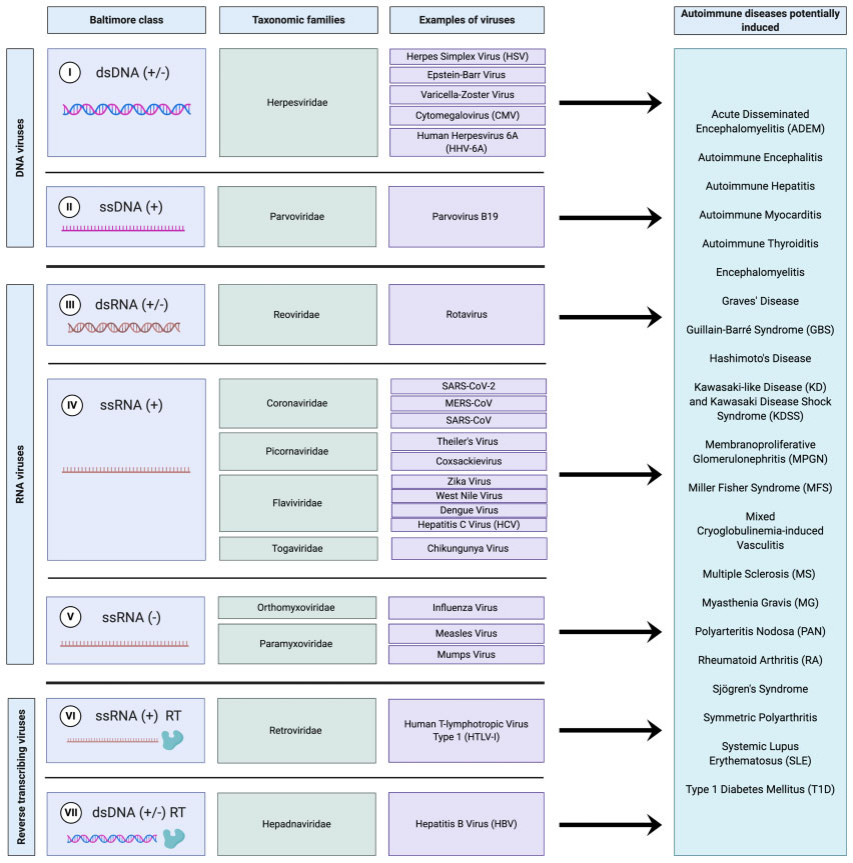
The development of a wide range of autoimmune diseases stemming from all seven Baltimore classifications of viruses. Baltimore classifications for viruses are from Mahmoudabadi and Phillips [80]. The development of RA after Chikungunya virus infection is described by Tanay [81], T1D after the mumps virus by Ramondetti *et al.* [82], Sjögren’s syndrome after HTLV-I by Quaresma *et al.* [83] and autoimmune diseases after HBV by Maya *et al.* [84]. The development of GBS after SARS-CoV-2 infection is described by Rahimi [85] and MFS and KD/KDSS after SARS-CoV-2 by Ehrenfeld *et al.* [39]. The development of all other autoimmune conditions after corresponding viral infections was recently summarized by Smatti *et al.* [86]

One of the most pronounced effects of widespread biota alteration on a society is the dramatic increase in the prevalence of autoimmune disease. Autoimmune diseases, now roughly 100 in number, affect >8% of the total US population. Unfortunately, as shown in Fig. 2, a common trigger for the induction of autoimmune disease is viral infection. Indeed, all major Baltimore classes of viruses, including major classes of RNA and DNA viruses, are thought to induce autoimmune disease in humans (Fig. 2). Thus, while exposure to SARS-CoV-2 may induce autoimmune disease [38–40] the virus is not unusual in this regard.

Another hallmark of high-income countries is the high prevalence of inflammation-associated neuropsychiatric disorders. For example, >1% of US women are affected by chronic fatigue syndrome [41, 42] and as many as 20% of US adults may be affected by major depressive disorder [43–45]. Neuropsychiatric disorders, like autoimmune diseases, are probably associated with biota alteration [26–29, 46–49]. Although the idea that viral infection can trigger neuropsychiatric disorders is difficult to assess, some evidence exists [50, 51]. Again, SARS-CoV-2 is possibly not unique in this regard, triggering a variety of neuropsychiatric issues including delirium, behavioral changes and encephalopathy [52, 53]. However, given limited data at the present time, it remains unknown whether SARS-CoV-2 can trigger long term neuropsychiatric issues.

Emerging evidence indicates that much of the morbidity/mortality associated with SARS-CoV-2 is in fact due to an overly aggressive immune response and ensuing cytokine storm [54–57] that may be partly autoimmune in nature [56]. In general, in the field of medicine, the presence of helminths is thought to produce an attenuated immune response due to secretion of immunoregulatory molecules [58, 59], creation of regulatory networks [10] and changes in mucosal surface permeability [60]. However, given that vertebrate immune function evolved in the presence of symbiotic helminths [34, 35], the field of medicine needs a change in perspective and may benefit greatly by considering the immune response in the presence of helminths to be ‘normal’ and function in the absence of helminths to be hyperresponsive [32, 59, 61]. With this view in mind, it seems intuitive that biota alteration may be, at least in part, responsible for part of the morbidity and mortality associated with SARS-CoV-2 in high-income countries.

## SYSTEMS HYGIENE AND COVID-19

The effects of hygiene on the COVID-19 pandemic are potentially complicated (Box 2). However, based on the effects of biota alteration on immune function and based on emerging data regarding the immune response to COVID-19 in those countries, one of us predicted in May of 2020 that COVID-19 would likely be less impactful in low-income countries than in high-income countries (Facebook post from Dr. William Parker’s Lab, last accessed December 17, 2020). This prediction was made despite the fact that social distancing and cessation of many business practices may be less feasible in low-income countries than in other countries. Emerging evidence now indicates that people living in areas with endemic exposure to helminths are in fact less impacted by infection with SARS-CoV-2 than are individuals living in high-income countries [2–6]. Numerous post-hoc explanations for this observation that surprised most infectious disease experts have been offered [2], including the presence of relatively fewer elderly and chronically ill individuals in low-income countries. While demographics apparently do account for some of the less severe effects of SARS-CoV-2 in low-income countries [6], the decreased levels of systems hygiene and autoimmune disease also appear to be associated with the reduced impact of SARS-CoV-2 in countries such as the Democratic Republic of Congo, the Republic of the Philippines and the Republic of Haiti [6, 62, 63].

Given the complexity of the pandemic, experts working in the field realize that the consensus at the present time is tenuous. In addition, current studies use measures of income, waterborne diseases, prevalence of autoimmunity or other factors that do not necessarily correlate exactly with the presence of helminths and may not be constant across a given country, potentially clouding the data. Nevertheless, if the initial conclusions drawn from early studies prove to be correct, it strongly supports the view that restoring the biota in high-income countries will not only decrease the burden of autoimmune and allergic disease but might also decrease the damage potentially caused by future pandemics of infectious disease.

## RESTORING THE BIOTA: WHERE WE ARE NOW

More than 50 years ago, Brian Greenwood deduced that high parasite burdens were probably responsible for very low levels of autoimmune disease in some communities [13] and subsequently demonstrated that exposure to a protist could in fact rescue laboratory mice from a lethal, lupus-like syndrome [15]. Importantly, Greenwood’s ‘protist therapy’ produced healthy mice with no apparent lingering side effects from the life-saving therapy, thus demonstrating that controlled exposure to a symbiont could in fact be highly beneficial. Although the laboratory mouse model used by Greenwood is still popular today [64], his cure did not garner interest from the scientific community. Similarly, studies in the 1970s by Preston [17] and Turton [18] showing that exposure to helminths eliminated seasonal allergies was never pursued by the medical community. It was not until about 2003, when Weinstock *et al.* [65–67] began controlled exposures of patients to the porcine whipworm in an effort to treat inflammatory bowel disease, that interest in developing a clinical therapy using symbionts picked up.

Studies evaluating the effects of protists and helminths on multiple sclerosis [23] suggest that a wide range of complex eukaryotic symbionts may achieve a similar therapeutic effect. However, since the reproduction of helminths is much more easily controlled than is the reproduction of protists, helminths rather than protists are preferred for development as a therapeutic. Systematic sociomedical studies evaluating the effects of helminth therapy on individuals engaging in self-treatment with helminths confirms studies in laboratory animals and indicates that the therapy is indeed very effective and safe for a range of common allergic, autoimmune and neuropsychiatric conditions [27, 28, 68].

Unfortunately, despite some ongoing work in the field using the human hookworm as a therapeutic agent, research is moving slowly without any unified effort, perhaps due to the same lack of attention that Greenwood’s work [69] faced more than half a century ago. Key problems with garnering interest include policy and regulatory issues that greatly diminish commercial incentives for developing naturally occurring symbionts as a therapy [70, 71]. In addition, the false bias that all helminths are harmful parasites tends to dissuade many from ever considering the idea [72]. Bias against helminths is understandable, but evidence suggests that considering all helminths to have the same effects on the human body would be the same as lumping salmonella and lactobacilli into a single group simply because they are both bacteria. For example, while some helminths can cause cancer [73], others have been shown to prevent cancer in animal models [74].

Another factor that has diminished interest in using helminths as a therapeutic agent is the unsubstantiated idea that helminth derived products rather than intact helminths might be effective therapeutics. This idea has garnered much interest because it promises financial rewards, but it seems highly unlikely that a single molecule, whether delivered via injection or by pill, could recapitulate the complex biological relationships between helminth and host that effectively modulate immune function [30]. Further, lack of interest in the field is likely encouraged by lackluster results of clinical studies that, although time consuming and costly, were probably not designed appropriately to evaluate the use of helminths as a therapeutic agent. For example, two primary concerns with prior studies are that (i) they do not take into account large variations in the amount of exposure to helminths necessary for beneficial effects in humans and (ii) they do not take into account potential nuances in the production and storage of living organisms that might affect their clinical efficacy [27, 75]. A further complicating problem is that the selection of helminth species for use in clinical trials thus far has been haphazard, dictated by convenience rather than by a systematic search for a benign helminth that effectively regulates immune function [76].

Perhaps the best indicator of impediments facing the use of naturally occurring organisms to effectively treat disease is the history of the fecal microbiota transplant. First established prior to 1960 as an effective means of treating a common but deadly condition known as recurrent *Clostridium difficile* colitis [77], the procedure did not become popular until 40 years later, only after tens of thousands of patients with recurrent *C. difficile* colitis died without the life-saving transplant [70]. Sadly, the transplant procedure is still not standard of care today, with unknown numbers of patients still dying from recurrent *C. difficile* colitis who might have been saved by fecal material from a healthy donor. This scenario highlights the problems currently facing helminth therapy and biota reconstitution in general. Without pharmaceutical-based incentives to drive medical practice, the modern medical enterprise falters. In essence, the concept of evolutionary mismatch applies: The system that evolved with a foundation of pharmaceutical involvement does not perform well when that underpinning is removed.

## CONCLUSIONS: PROBLEMS AND SOLUTIONS

Independent lines of evidence, including epidemiologic studies, studies using animal models and clinical observations, point to the idea that we need exposure to helminths in order to avoid biota alteration and immune hypersensitivity. Further, prior studies indicate that our response to acute viral infections may be exacerbated by biota alteration, and emerging evidence suggests that this paradigm also applies to the immune response against SARS-CoV-2. Although vaccines against SARS-CoV-2 are forthcoming, the virus may become endemic and seasonal [78], providing further impetus for efforts aimed at reversing the effects of biota alteration in high-income countries. In addition, effective immune function is undoubtedly important when considering current pandemics of autoimmune disease, allergy, neuropsychiatric disorders and susceptibility to as yet unknown pandemics induced by viral infections that might occur in the future.

At the same time, eliminating systems hygiene to alleviate the effects of biota alteration would induce unacceptable pandemics of infectious disease. Eliminating personal hygiene would exacerbate current problems with infectious disease, increase exposure to agents that trigger chronic disease and utterly fail to relieve the adverse effects of biota alteration.

Fortunately, a course of action is available (Box 3). Clinical trials need to be conducted to determine how to alleviate the adverse effects of biota alteration. Helminths, like bacteria, are not all pathogens or parasites [31, 76], despite our prejudice against them. Indeed, benign helminths are already available which can be evaluated for their potential to reverse the effects of biota alteration [30, 68, 76, 79]. Unfortunately, clinical trials with ‘helminth therapy’ to date have been problematic, not taking into account nuances with the cultivation of helminths, selection of helminths with the best benefit-to-risk ratios and the wide range of individual-to-individual variation in the effective treatment regimens [27, 75]. Action is urgently needed given the importance of immune function, not only for immunological tolerance to our own bodies and harmless environmental antigens, but also for effective, self-preserving responses against current and future pathogens. Unfortunately, logical and reasonable courses of action, summarized in Box 3, have largely been hampered by current regulatory pathways and financial incentives that encourage drug development but impede more straight-forward reversal of evolutionary mismatches such as biota alteration [69–71]. With this in mind, questions regarding the future of immune function in high-income countries (Box 4) point toward much needed changes in policy and regulation to incentivize work on restoration of a healthy biota.

Box 1. Clearing the cloud of confusion surrounding hygiene: two types of hygiene with different effects on COVID-19 and on our immune system

1. The term hygiene can refer to either one of two very different ways of avoiding infection. The types of hygiene, labeled here as personal hygiene and systems hygiene, have very different effects on humans.
2. Personal hygiene, including hand washing and social distancing, helps prevent transmission of many infectious diseases, including COVID-19 and the flu. Diseases prevented by personal hygiene are often dangerous and detrimental to immune function.
3. Improved personal hygiene can reduce exposure to a range of factors that can trigger chronic inflammatory conditions. Such inflammation-inducing factors include mold, insect-derived allergens and acute infections from a wide range of viruses that includes SARS-CoV-2.
4. Systems hygiene is the implementation of modern sanitation (water treatment plants, sewage systems, indoor plumbing) and food processing and storage technology. Systems hygiene, like personal hygiene, also helps prevent transmission of many infectious diseases that are unhelpful for immune function and detrimental to health. For example, systems hygiene prevents pandemics of typhoid, cholera and amoebic dysentery.
5. Systems hygiene, however, causes a virtually complete loss of some types of organisms that have lived in the bodies of our ancestors for hundreds of millions of years. Intestinal worms, called helminths, are one of the key species that have been all but driven extinct by systems hygiene in modern society. Even though many helminth species are harmful parasites, others cause little or no disease. Importantly, scientific evidence demonstrates that exposure to helminths appears to be necessary for healthy immune function and that their absence leaves us susceptible to pandemics of chronic, non-infectious inflammatory diseases. Such diseases include allergy, autoimmunity and probably neuropsychiatric issues such as major depression, anxiety disorders and chronic fatigue syndrome.
6. Reductions in personal hygiene cannot reverse the detrimental loss of helminths caused by systems hygiene. Rather, reductions in personal hygiene would only cause more infectious diseases such as COVID-19 and would result in more exposure to inflammation-inducing factors, all of which would be harmful.
7. We cannot live without systems hygiene. Loss of systems hygiene in modern society would, in theory, reverse the detrimental loss of helminths caused by that type of hygiene. However, the resulting waves of deadly, infectious diseases would be catastrophic.

Box 2. The complex relationship between hygiene and COVID-19

1. Personal hygiene (social distancing, handwashing and wearing a mask that covers the mouth and nose) is the best approach to avoiding SARS-CoV-2 and the devastating effects of the COVID-19 pandemic.
2. Systems hygiene (sewer systems, water treatment systems and food processing and storage facilities) reduces or even eliminates exposure to intestinal worms, called helminths. However, emerging evidence suggests that human populations with helminths are less prone to have deadly or severe adverse reactions to SARS-CoV-2.
3. Many viruses, including SARS-CoV-2, can trigger autoimmune reactions and/or diseases. Nevertheless, it is the loss of helminths in the body that predisposes the immune system to develop autoimmune diseases and allergic disorders.
4. High-income countries are stuck between a hygiene rock and a hygienic hard place: they need controlled exposures to helminths to ensure effective immune function, but at the same time they need effective systems hygiene to avoid environmental exposures to deadly organisms such as typhoid or cholera.

Box 3. The path forward for medicine: how we can avoid infectious disease and still obtain enough environmental exposures to support our immune system?

1. The detrimental effects of the loss of intestinal worms, called helminths, caused by factors such as toilets and water treatment facilities can be readily reversed by domestication of select helminth species and artificial enrichment of the human body with those organisms. Considerable evidence supports the view that such an effort would greatly reduce the burden of allergy, autoimmunity and probably neuropsychiatric disorders currently experienced in high-income countries. It also seems very likely that these efforts would decrease the likelihood of having adverse reactions to infections with a wide range of viruses, including SARS-CoV-2.
2. Work in the field of helminth therapy indicates that current trials based on pharmaceutical models fail to take into account critical issues, including individual-to-individual variation in the effective dose, risk/benefit ratios when selecting helminth species and the importance of specific husbandry conditions when cultivating and preserving helminths. Trials need to be conducted with appropriate methods of production of helminths, dosing regimens designed for helminth therapy and rational selection of helminth species.
3. Although benign helminths are currently available for human testing, interest in conducting clinical trials is hampered by high costs and intellectual property issues. Given that therapies based on naturally occurring organisms cannot be patented, financial incentives for moving forward are lacking.
4. In general, governments and research organizations need to focus on disease prevention by dealing with evolutionary mismatches rather than treatment of disease solely using pharmaceutical approaches. This principle applies to many facets of modern medicine, including how we deal with the adverse effects of the loss of helminths on our immune system.

Box 4. Frequently asked questions regarding COVID-19 and hygiene

1. Will one or two years of social distancing for COVID-19 ‘crash our immune system’? No. The loss of particular organisms from within our bodies due to widespread use of sewage treatment facilities and water purification plants does indeed damage immune function and could lead to chronic inflammatory diseases and probably adverse reactions to viruses such as SARS-CoV-2. However, reducing personal hygiene would have no effect on this problem.
2. Is it possible to restore specific lost species in our body and simultaneously maintain hygiene to avoid pandemics of infectious disease? The answer is absolutely yes—in theory. There is no evidence to suggest that we need to be exposed to disease causing organisms to have the appropriate array of organisms in our body for healthy immune function. We can domesticate the organisms we need and introduce them artificially. Long-standing evidence indicates that exposure to selected intestinal worms will be effective at reducing disease without causing health problems, if that solution can be implemented.
3. Why haven’t we already corrected the problem by restoring organisms that have been lost? The answer to this question is multifaceted. First, even though the adverse effects of missing particular organisms have been mounting for over a century, we only recently understood the problem well enough to do something about it. Second, unfortunately, organisms such as intestinal worms, called helminths, that could be used to restore the ecosystem of the body, are classified as drugs under current regulations. Classification as a drug presents a number of problems, including a drug development process unsuited for naturally occurring organisms such as helminths. Third, the limited trials that have taken place thus far using helminths to restore the biodiversity of the body did not take into account several important factors that need to be considered when designing trials using helminths as therapeutic agents and thus did not move the field forward.
4. How long will it take to restore the needed species in our bodies? The answer is that, since candidate helminths have already been identified and are currently present in the environment without causing disease, products for consumer use could be available within a year or two of the initiation of well-designed trials. However, we do not yet know when policy makers and health officials will address the issues that are currently impeding research in this area. Thus, the technology is available to solve the problem quickly, but it is difficult to predict when necessary policy and funding changes will be made.
5. What about the gut microbiota, the bacteria? Can we use bacteria to help restore healthy immune function to our bodies? The answer is unknown. Our diets are the major drivers of the microbiota community composition, and we don’t know if it’s possible to fix our microbiota without fixing our diets. Further, we do not know how important alterations in the microbiota are in terms of a causative agent of chronic inflammatory disease.
